# Triple negative aggressive phenotype controlled by miR-135b and miR-365: new theranostics candidates

**DOI:** 10.1038/s41598-021-85746-w

**Published:** 2021-03-22

**Authors:** Gloria Bertoli, Claudia Cava, Fabio Corsi, Francesca Piccotti, Cristina Martelli, Luisa Ottobrini, Valentina Vaira, Isabella Castiglioni

**Affiliations:** 1 Institute of Molecular Bioimaging and Physiology, National Research Council (IBFM-CNR), Via F.Cervi 93, 20090 Segrate-Milan, Milan, Italy; 2 Department of Biomedical and Clinical Sciences “Luigi Sacco”, University of Milan, Milan, Italy; 3 Breast Unit, Department of Surgery, Istituti Clinici Scientifici Maugeri IRCCS, Pavia, Italy; 4 Nanomedicine and Molecular Imaging Lab, Istituti Clinici Scientifici Maugeri IRCCS, Pavia, Italy; 5 Deparment of Pathophysiology and Transplantation, University of Milan, Milan, Italy; 6 Division of Pathology, Fondazione IRCCS Ca’ Granda Ospedale Maggiore Policlinico, Milan, Italy; 7 University of Milan-Bicocca, Piazza della Scienza 3, 20126 Milan, Italy

**Keywords:** Cancer, Breast cancer, Mechanisms of disease, Cancer epigenetics, Gene regulatory networks, miRNAs

## Abstract

Triple negative breast cancer (TNBC) accounts for about a fifth of all breast cancers and includes a diverse group of cancers. The heterogeneity of TNBC and the lack of target receptors on the cell surface make it difficult to develop specific therapeutic treatments. These aspects cause the high negative prognosis of patients with this type of tumor. The analysis of the molecular profiles of TNBC samples has allowed a better characterization of this tumor, supporting the search for new reliable diagnostic markers. To this end, we have developed a bioinformatic approach to integrate networks of genes differentially expressed in basal breast cancer compared to healthy tissues, with miRNAs able to regulate their expression. We studied the role of these miRNAs in TNBC subtype cell lines. We therefore identified two miRNAs, namely miR-135b and miR-365, with a central role in regulating the altered functional pathways in basal breast cancer. These two miRNAs are differentially expressed in human TNBC immunohistochemistry-selected tissues, and their modulation has been shown to play a role in the proliferation of tumor control and its migratory and invasive capacity in TNBC subtype cell lines. From the perspective of personalized medicine, we managed to modulate the expression of the two miRNAs in organotypic cultures, suggesting their possible use as diagnostic and therapeutic molecules. miR-135b and miR-365 have a key role in TNBC, controlling proliferation and invasion. Their detection could be helpful in TNBC diagnosis, while their modulation could become a new therapeutic tool for TNBC.

## Introduction

Triple-negative breast cancer (TNBC) is one of the four molecular immunohistochemical subtypes of breast cancer (BC) described in comparison to normal epithelium^1^. It represents a percentage of 10–20% of all BC tumors^2^. The overall 5-year survival rate for TNBC is 50–60%. The recurrence peak occurs within the first 3–5 years from diagnosis. On the contrary, late recurrences decline over the following 5 years^3^.

What is described by immunohistochemistry (IHC) as TNBC subtype, defined by negative immunohistochemical staining for estrogen receptor (ER) and progesterone receptor (PR) and lack of human epidermal growth factor receptor 2 (Her2/*neu*) overexpression^4^, includes a heterogeneous group of tumors from a genetic point of view, when compared to both normal tissue and other types of BC^5^. The classification given by the IHC analysis is limited to the description of the tumor, based on the presence on the cell membrane of hormone receptors (ER, Pr and HER2). The molecular classification, analyzing the expression level of the mRNAs, indicates the triple negative subtype with the name of basal (see^6^), which is characterized by the expression mainly of cytokeratin 5/6, 14 and 17, of laminin and of lipid protein binding 2^7^.

However, a single set of markers that specifically describes basal BC does not yet exist. Clustering algorithms applied on molecular profiles are able to find further subclasses also in the basal subtype, but these subcategorizations require a greater number of samples to be better defined. Moreover, numerous variables could affect the subcategories of the basal subtype, drawing attention to the need of a standardized method and substantially large databases to define molecular classes of basal breast cancer. Nevertheless, there is a good degree of overlap between the molecular definition of basal and the description of TNBC defined by the IHC (about 80%)^8^. This also justifies the fact that both classifications are able to predict the worse prognosis of this tumor subtype, when they encounter it: the basal subtype, as well as TNBC, are characterized by high histological grade, high mitotic index and low differentiation and are often associated with the presence of metastases and lymph node involvement^9^.

The higher heterogeneity of this tumor type and the lack of IHC target receptor (ER, PR, HER2) resulted in the absence of FDA-approved target therapies for TNBC^10^. Because of the poor prognosis observed in TNBC patients and the lack of approved therapeutic treatment (see Sacituzumab/govitecan approval for TNBC from FDA, May 2020), this subtype accounts for a disproportionate number of metastatic cases and breast cancer deaths.

The possibility of using molecular profiling to better characterize and diagnose the TNBC subtype with higher accuracy would allow the comprehension of the altered molecular mechanisms in this specific subtype of BC.

**Figure 1:**
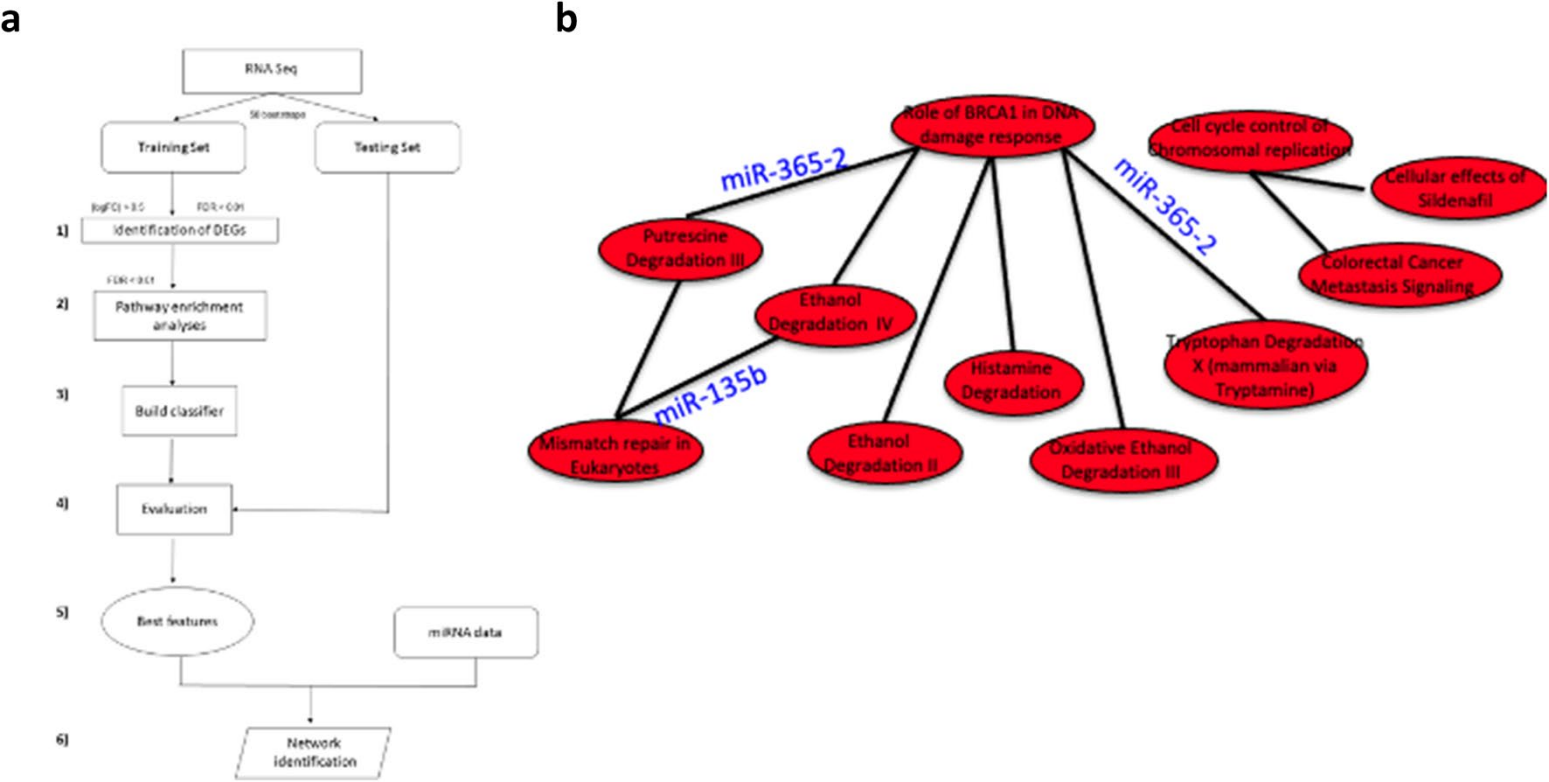
Overview of the data, methods (**a**) and results (**b**) for TBNC network identification (**A**) Workflow of the proposed computational approach. It consists of 6 steps. (1) identification of differentially expressed genes (DEGs) between basal and normal samples from TCGA data, (2) pathway enrichment analysis. In this step we identified the pathways enriched with DEGs, (3) construction of a classifier to distinguish basal versus normal samples using a metric able to reveal the interactions among pathways, (4) the classifier was evaluated using Area Under Curve (AUC) in the training and testing test, (5) we selected the pairs of pathways that achieved the best AUC values, (6) we applied an integration algorithm between miRNA and pathway in order to identify miRNAs regulating pathway network in basal breast cancer. (**B**) miRNAs regulating pathway network. The figure shows the top 10 pairs of pathways that achieved the best performance in the classification of basal versus normal samples and miRNAs regulating this network.

In order to identify new possible epigenetic molecules able to diagnose molecularly TNBC, we developed an in silico approach that identifies differentially expressed genes (DEGs) of basal BC compared to normal tissues; this information is integrated with functional pathways, and biological relationships among cellular components (network), also taking into account the microRNAs’ (miRNAs) regulatory role on the network (Fig. 1a). Indeed, miRNAs are small non-coding RNA with a post-transcriptional regulatory role on several mRNAs involved in coding proteins performing cancer hallmarks’ functions^11,12^. With this approach, we identified a small group of miRNAs able to control differentially expressed mRNA, specifically altered in basal BC, involved in specific functions of the tumor network. As miRNAs are cell- and tumor specific^13^, and as each miRNA belongs to a complex network of interactions, the alteration of miRNA expression levels could severely impact on the target mRNAs and, consequently, on the tumor behavior. In this study, we characterized the biological role of TNBC-associated miRNAs and found that *Hsa-miR-135b* (miR-135b) and *Hsa-miR-365* (miR-365) have diagnostic properties, being able to classify IHC-selected TNBC samples versus normal tissues.

Furthermore, their altered expression, i.e. the increase of miR-135b and the decrease of miR-365, supported the aggressive, metastatic phenotype of TNBC cells, controlling their proliferation, migration and invasion. The efficacy of transfection of synthetic oligonucleotide in the cells could suggest their potential use as possible therapeutic agent.

## Results

### Computational approach

The computational analysis, performed to identify the pathways to which the basal-descriptive genes belong to and to integrate this information into a functional network, consists of 6 steps (Fig. 1a). In the first step, we identified and selected a set of DEGs between basal and NS; in the second step, we identified pathways enriched with DEGs; in the third step, we generated a classifier to distinguish basal versus NS using the pathway metric.

The first three analyses (identification of DEG, pathway enrichment analysis and the classification) were repeated 50 times on different generated training dataset.

In the fourth and fifth step, the classifier was evaluated with performance indices and the features that obtained the best results are selected and tested on the testing dataset. In the last step we applied an integration algorithm between miRNA and pathway network in order to identify a basal BC network consisting of miRNAs regulating a pathway network.

### Step 1: Identification of differentially expressed genes

In order to identify differentially expressed genes between basal and NS, a statistical analysis using TCGA biolinks package was applied^14^, considering a gene differentially expressed when its |logFC|> 1 and *p* value < 0.01. The *p* values were adjusted by the Benjamin–Hochberg method for multiple testing corrections^15^^.^

### Step 2: Pathway enrichment analysis

Once the DEGs were identified, the pathway enrichment algorithm was applied to verify in which of the 589 pathways from the Ingenuity Pathway Analysis (IPA) database the DEGs were inserted. The algorithm was performed using the Fisher's Exact Test; we considered a pathway to be enriched with DEGs if the associated adjusted *p* value was < 0.01.

### Step 3: Build the classifier

The strength of the interactions among pathways were indicated by calculating a discriminating score, as reported in^16^. This score, which indicates the relationship between pairs of pathways, was calculated among all possible pairs of pathways enriched by the differentially expressed genes.

A machine-learning model (Random Forest classification) was developed using this score for each sample in order to build a network able to classify basal and NS.

### Step 4 and 5: Evaluation and features selection

AUC was estimated as performance index. We considered for each of the 50 bootstraps the top 10 pairs of pathways that achieved the best performance in the training dataset. The testing dataset was used to validate the top 10 pairs of pathways obtained in the training step. At the end of all 50 bootstraps we selected the best top 10 pairs of pathways.

### Step 6: Network identification

Once the network consisted of 10 pairs of pathways was obtained, by applying the mutual information and Fisher's exact test we identified those miRNAs that are able to regulate a significant number of genes in the pathway network. To each of these miRNAs we associated an index, namely the degree centrality (DC): miRNAs with higher DC (HDC miRNAs) were those that regulate the highest number of genes within functional coupled-pathways.

### miR-365 and miR-135b have a high degree centrality in TNBC

The couple pathways that achieved the best performance in the classification of basal *versus* normal samples (NS) were listed in Fig. 1b. The interactions among pathways were identified by a discriminating score able to distinguish with the best performances basal versus NS, while miRNAs regulating the network were obtained in the last step of computational approach using mutual information and Fisher's exact test.

Ethanol degradation, DNA damage response, mismatch repair and cell cycle are the main functions emerging within the network. Indeed, in both training and testing dataset the network obtained a AUC with a median > 95%.

The in silico analysis revealed that 2 miRNAs are altered in TNBC, miR-365 and miR-135b with a key role in the network (Table 1). A schematic view of the regulatory interaction among miRNAs and coupled pathways is depicted in Fig. 1b. In particular, miR-365 has a degree centrality of 42, controlling 42 over 96 genes within the 2 couple pathways (Putrescin degradation, Role of BRCA1 in DNA damage response and Tryptophan degradation X), while miR-135b has a degree centrality of 12, controlling 12 over 35 genes within 1 couple pathways (Ethanol degradation X and Mismatch repair in eukaryotes).

**Table 1:** List of genes controlled in coupled pathways by the selected two miRNAs.

Pairwise pathway	miRNAs	Genes in pathway 1	Genes in pathway 2
1. Ethanol degradation;	Hsa-miR-135b (predicted Up)	*ACSL1*/*3*,* ALDH1A1*/*1B1*/*3A24A1*	*FEN1*,* MSH6*,* RFC2*,* RFC4*,* RPA1*,* SLC19A1*
2. Mismatch repair			
1. Role of BRCA1 in DNA Damage Response	Hsa-miR-365–2 (predicted Down)	*ATR*,* CHEK2*,* E2F1*,* MLH1*,* FANCA*/*B*/*C*/*E*,* MSH6*,* NBN*,* PLK1*,* RB1*,* RBBP8*,* RBL1*,* RFC5*	*ALDH1A1*/*1A3*/*2*,* IL4I1*,* MAOA*,* SMOX*
2. Putrescine Degradation III			
1. Tryptophan Degradation X	Hsa-miR-365–2 (predicted Down)	*ALDH1A1*/*1A3*/*2*,* IL4I1*,* MAOA*,* SMOX*	*ATR*,* CHEK2*,* E2F1*,* FANCA*,* FANCB*,* FANCC*,* FANCE*,* MLH1*,* MSH6*,* NBN*,* PLK1*,* RB1*,* RBBP8*,* RBL1*,* RFC5*
2. Role of BRCA1 in DNA Damage response			

Regarding the expression levels of these two miRNAs, in silico analysis suggested that miR-365 is downregulated in basal tumor cells compared to normal tissue^17^, while miR-135b is upregulated in basal tumor cells compared to normal tissue^18^.

### miR-135b and miR-365 target analysis

Looking in more depth to the genes controlled by these two miRNAs in each pathway using mutual information, we identified *ALDH* family members (*ALDH1A1*/*1A3*, *ALDH2*),* IL4I1*,* MAOA*,* SMOX* belonging to ‘Tryptophane degradation X’ and *ATR*,* CHEK2*,* E2F1*,* FANC* family members (*FANCA*/*B*/*C*/*E*),* MLH1*,* MSH6*,* NBN*,* PLK1*,* RB1*,* RBBP8*,* RBL1*,* RFC5* belonging to *‘*Role of BRCA1 in DNA damage response’ as possible targets of miR-365, and *ACSL1*,* ACSL3*,* ALDH1A1*/*1B1*/*3A2*/*4A1* belonging to *‘*Ethanol degradation IV’ and *FEN1*,* MSH6*,* RFC2*,* RFC4*,* RPA1*,* SLC19A1* belonging to *‘*Mismatch repair in eukaryotes’ as possible targets of miR-135b (Table 2).

**Table 2:** Immunohistochemical characterization of TNBC human samples.

Sample	% ER	% Pr	% Ki67	cerb B2
#1	0	0	40	0
#2	0	0	70	1 +
#3	0	0	60	0
#4	0	0	60	0
#5	0	0	15	0
#6	0	0	5	1 +
#7	0	0	25	0
#8	0	0	8	0

### miR-135b and miR-365 are differentially expressed in basal cell line

**Figure 2:**
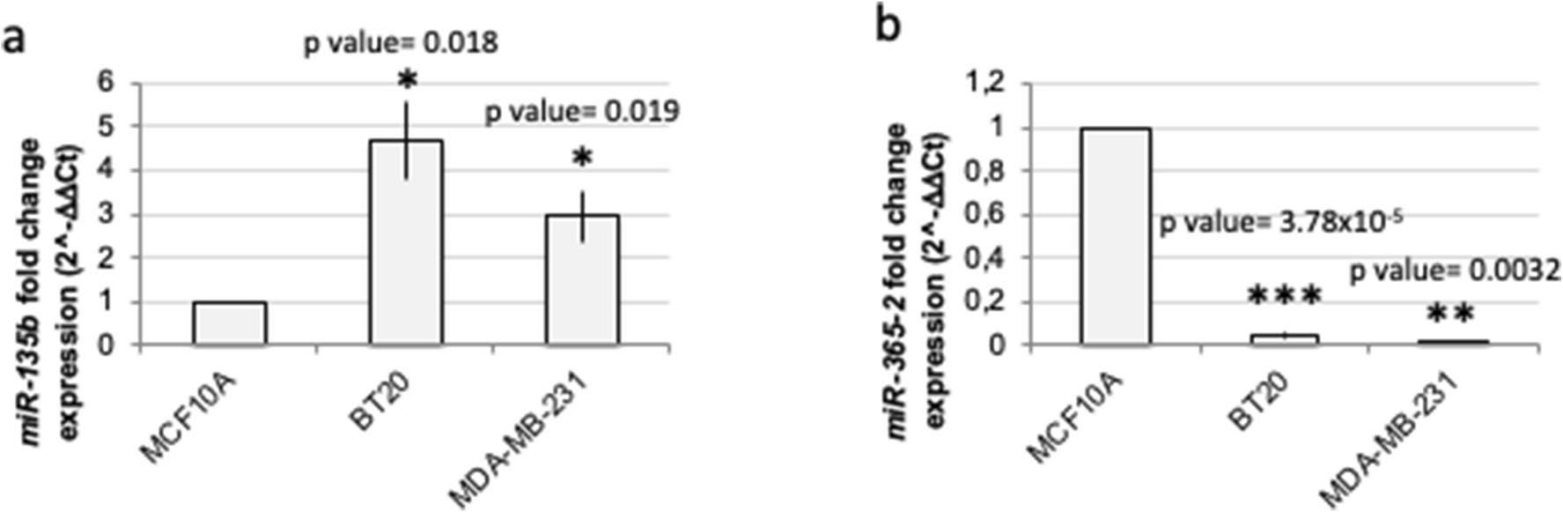
Validation of miRNA expression in MDA-MB-231 basal cell line model. *miR-135b* (**a**), and *miR-365-2* (**b**) expression levels were evaluated by RT-qPCR analysis in BT20 and MDA-MB-231 cell lines compared with normal-like MCF10A cells. The results are the average of three independent experiments. T test *p* value < 0.05 (*), 0.01 (**), 0.001 (***).

The in silico analysis predicted miR-135b upregulation and miR-365 downregulation in basal breast cancer compared to normal tissue. To validate the level of expression of these two miRNAs, we performed a RT-qPCR analysis of both miRNAs in two TNBC cell lines, namely BT20 and MDA-MB-231, compared to MCF10A (Fig. 2). The results confirm miR-135b upregulation in BT20 and MDA-MB-231 compared to MCF10A normal-like cell line, with fold change values of 4.70 ± 0.89 in BT20, 2.93 ± 0.56 in MDA-MB-231 compared to MCF10A normal-like cell line (t test *p* value = 0.018 in BT20 and 0.019 in MDA-MB-231 compared to MCF10A, respectively). RT-qPCR analysis confirmed also miR-365 downregulation in BT20 and MDA-MB-231, compared to MCF10A normal-like cell line, with fold change values of 0.044 ± 0.010 in BT20, 0.011 ± 0.007 in MDA-MB-231, respectively (t test *p* value = 3.78 × 10^–5^ in BT20 and *p* value = 0.0032 in MDA-MB-231 compared to MCF10A). A further confirm was obtained by in silico analysis of different GEO datasets on several cell lines (supplementary Figures 1–3).

### miR-135b and miR-365 control cell proliferation

**Figure 3:**
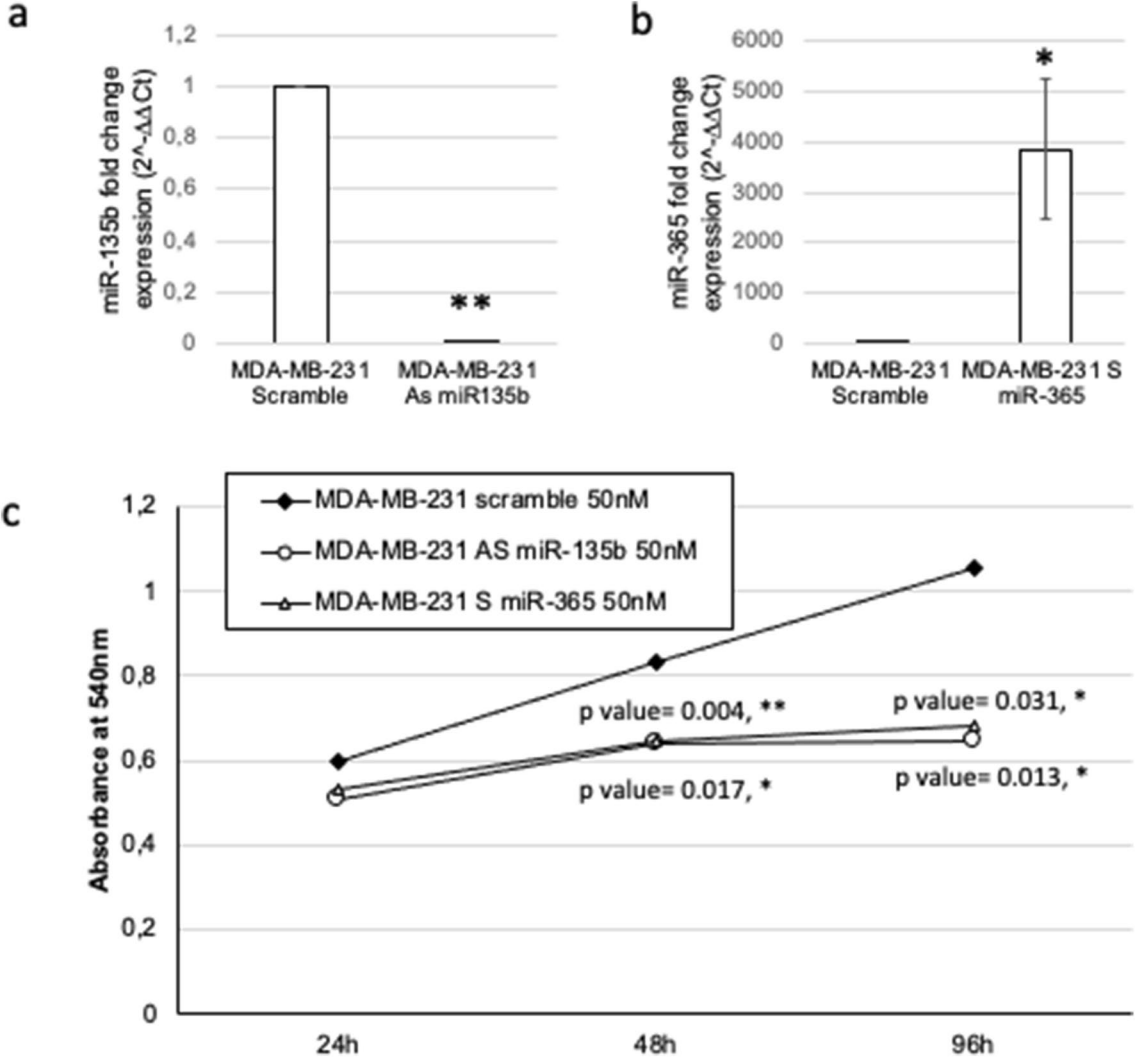
Proliferation of the cells after miRNA modulation. MTT assay (Absorbance at 540 nm) revealed that the MDA-MB-231 treated with 50 nM As miR-135b (**a**) or S miR-365 (**b**) reduced significantly the rate of proliferation (**c**). The graph represent the average of the absorbance at 540 nm of three counts performed in duplicate experiments (T test compared to untreated cells, *p* value < 0.05, *).

As several of the putative identified targets of miR-135b and miR-365 have a role in cell proliferation, such as Checkpoint Kinase 2 (*CHEK2*)^19^, E2F Transcription Factor 1 (*E2F1*)^20^, the tumor suppressor retinoblastoma protein (*RB1*)^21^, or the genes of the FANC family^22^, we first analysed the effect of miR-365 upregulation or miR-135b suppression on proliferation of the cells. In order to perform the in vitro experiment on cell lines, we used MDA-MB-231 cells as they grow faster than BT20 cells. After confirming that the treatment with 50 nM As miR-135b or S miR-365 affected miRNAs’ expression compared to scramble treated cells (Fig. 3a,b, respectively), we observed that both treatments were able to slow down proliferation by MTT assay in a significant way at 48 and 72 h, in respect to scramble-treated cells (Fig. 3c).

### miR-135b and miR-365 control cell migration and invasion

Among the possible target identified with in silico analysis, we found some genes that are involved in DNA repair^23^ (i.e., Ataxia Telangiectasia And Rad3-Related Protein *ATR*), migration (*E2F1*)^24^ or invasion (i.e., Replication factor C5, *RFC5*)^25^.

**Figure 4:**
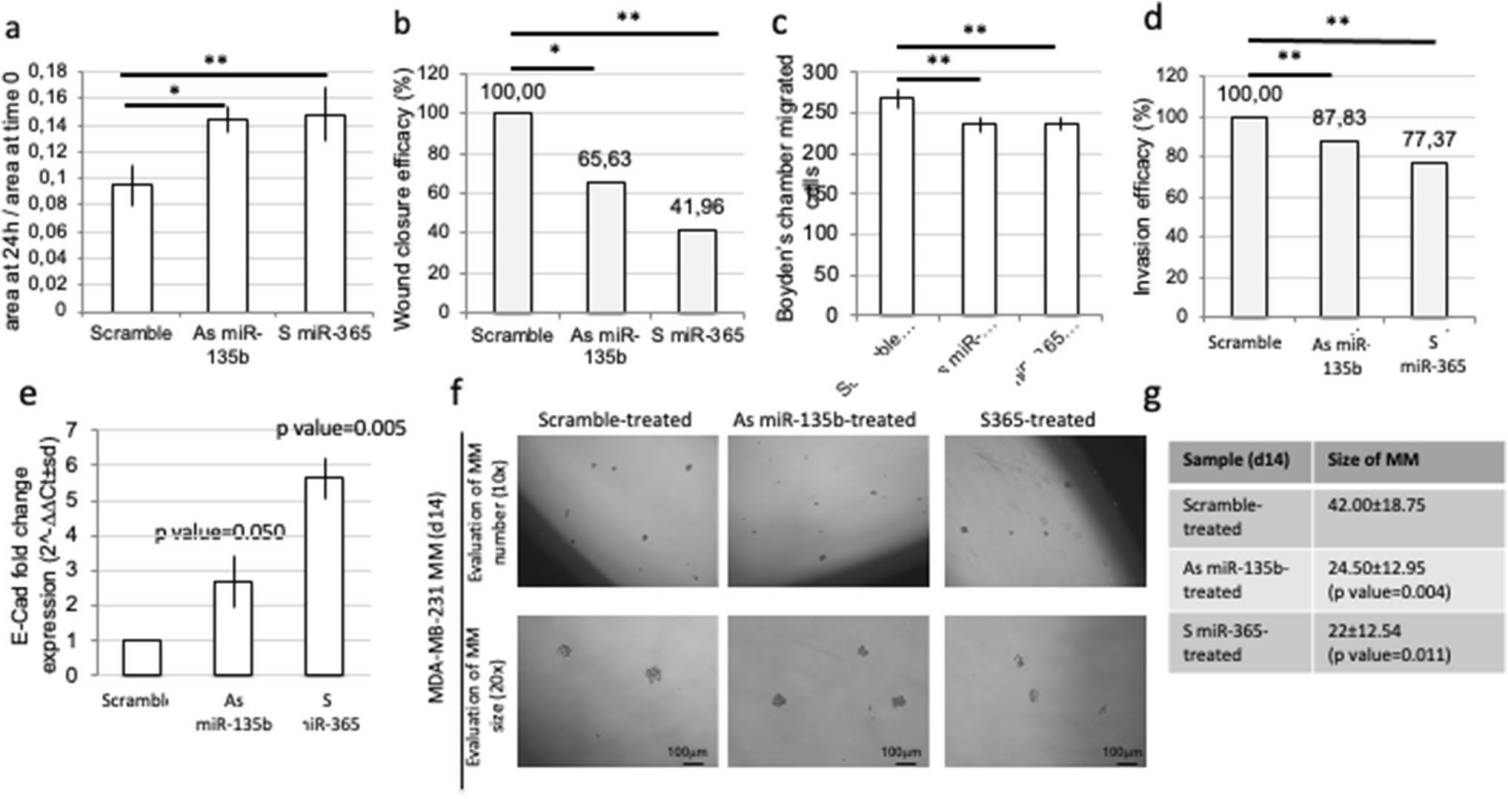
miR-135b and miR-365 are able to control cell migration, invasion and MET. (**a**) Result of the wound healing assay revealed that both reduction of miR-135b or induction of miR-365 reduced the capability of MDA-MB-231 cells to close the wound. The area of wounds at 24 h normalized on the same area at 0 h was measured for 50 nM scramble-treated, 50 nM As miR-135b-treated and 50 nM S miR-365 treated cells and compared to that of untreated cells. The measures (average ± sd) were performed in 4 different point of the wound, and the experiment was performed in duplicate (T test compared to scramble-treated cells, *p* value < 0.05, *; < 0.01, **). (**b**) The percentage of wound closure efficacy has been calculate for each sample. (**c**) The counts of MDA-MB-231 cells, migrated through the Boyden chambers, were taken after 24 h of treatment with 100 nM scramble, As *miR-135b* or S *miR-365* oligonucleotides. Data were normalized on scramble treated cells. The bars are the average ± sd of three counts performed in duplicate experiments (T test, *p* value < 0.05*; *p* value < 0.01 **). (**d**) The percentage of invasion efficacy is also calculated compared to scramble-treated cells. (**e**,**f**) The MDA-MB-231 treated with As miR-135b or S miR-365 oligonucleotides (50 nM) for 72 h showed significant increased levels of E-cadherin (E-Cad, epithelial marker) (**e**) and a reduced capability of MS formation (**f**). Reduction of average MS size after 50nMtreatment of As miR-135b or S miR-365 for 14d was quantified. Average of surface MS size ± sd is indicated in the table (t test versus scramble-treated cells, *p* value is indicated) (**g**).

We tested if the modulation of miR-135b or miR-365 was able to affect the MDA-MB-231 cell migration, by wound healing test. The treatment with As miR-135b or S miR-365 on MDA-MB-231 cells decreased their ability to fill up the wound, as shown by the increase of the ratio between wound area at time 24 h/wound area at time 0 h (Fig. 4a), with a percentage reduction of 35% for As miR-135b (t test *p* value = 0.035, *) and of 59% for S miR-365 (t test, *p* value = 0.001,**) in respect to scramble-treated cells (Fig. 4b).

To test the ability of miR-135b and miR-365 to control the invasion ability of the cells, we performed the Boyden’s chamber test. The treatment with 50 nM of As miRNA-135b or S miR-365 decrease significantly the capability of the cells to heal the wound (Fig. 4c). In particular, the modulation of both miRNAs reduced the invasion ability of the cells of about 13.2% for As miR-135b (t test, *p* value = 0.003, **) and of 22.7% for S miR-365 (t test *p* value = 0.003, **) in respect to untreated cells (Fig. 4d).

### miR-135b reduction induced mesenchymal-to-epithelial transition (MET)

The reduction of miR-135b expression, obtained by As treatment, or the increase of miR-365, obtained by mimic (S) treatment, lead to increased in E-Cadherin expression (Fig. 4e). Moreover, As miR-135b and S miR-365 treatments reduced the ability of the cells to form mammospheres (MS), compared to both untreated or scramble treated cells (Fig. 4f). The reduced ability of MS formation considers the size of the MS (Fig. 4g).

### miR-135b and miR-365 are differentially expressed in human sample

**Figure 5:**
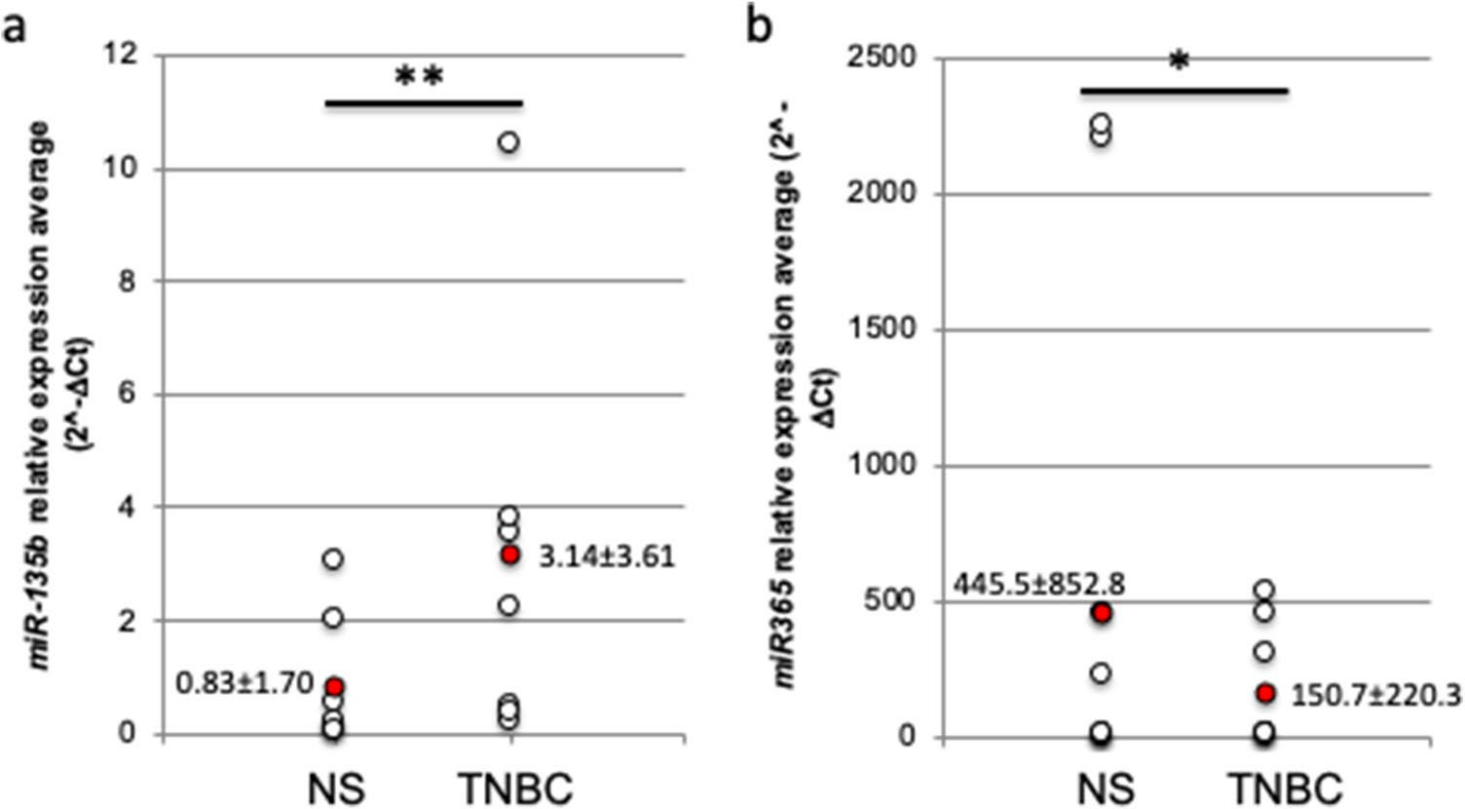
*miR-135b* and *miR-365* are differentially expressed in human basal BC samples. *miR-135b* (**a**), and *miR-365* (**b**) expression levels were evaluated by RT-qPCR analysis in human basal BC (TNBC) or surrounding healthy tissue samples (normal samples, NS). The average of three independent experiments is depicted in red. T test *p* value < 0.05 (*), 0.01 (**).

The RT-qPCR analysis revealed that also in human TNBC, miR-135b relative expression is upregulated (Fig. 5a; values of 3.14 ± 3.61 in TNBC tissues vs. 0.83 ± 1.70 in normal samples) and miR-365 relative expression is downregulated (Fig. 5b; values of 150.7 ± 220.3 in TNBC tissues vs 445.5 ± 852.8 in normal samples) compared to normal surrounding tissues. An in silico validation was performed on independent GEO datasets, GSE38167 and GSE58606. The results confirm the upregulation of miR135b and the downregulation of miR-165 in TNBC compared to normal tissues (see supplementary Figure 2).

### In vitro treatment on organotypic TNBC cell culture is effective in modulating miRNAs expression

**Figure 6:**
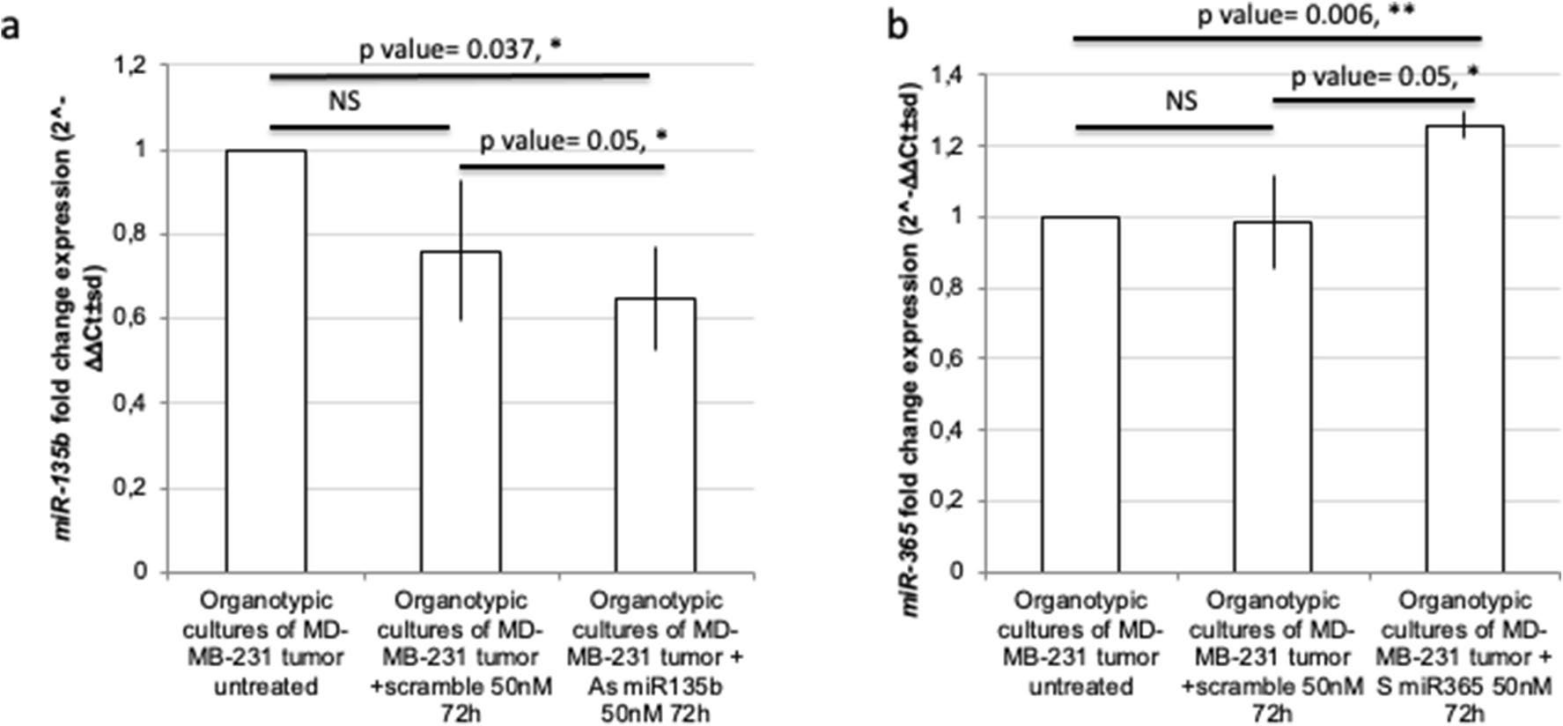
Therapeutic treatment with miR-135b and miR-365 oligonucleotides in MDA-MB-231 organoids modulates their expression. *miR-135b* (**a**), and *miR-365* (**b**) fold change expression levels were evaluated by RT-qPCR analysis in human organotypic-like cultured slices obtained from MDA-MB-231 tumors, in vitro treated with 50 nM 72 h of As *miR-135b* or S *miR-365* oligonucleotides. The average of two independent experiments is depicted. T test, *p* value < 0.05(*), 0.01 (**).

In order to evaluate the efficacy of oligonucleotide treatment in in vitro cultured organotypic slices of tumor, mimicking an organotypic-like culture, and to propose this treatment as a possible tool for TNBC therapy, we treated MDA-MB-231 tumor slices in vitro for 72 h with 50 nM As miR-135b or S miR-365 oligonucleotides. RT-qPCR analysis revealed that indeed the As oligonucleotide treatment was effective in reducing miR-135b (the fold change miR-135b expression was 0.67 ± 0.12 in As miR-135-treated tumor culture, compared to 0.76 ± 0.16 in scrambled-treated group) (Fig. 6a; t test *p* value = 0.05 compared to scramble and 0.037 compared to untreated organotypic-like culture) and the S oligonucleotide treatment induced miR-365 expression (Fig. 6b; the fold change miR-365 expression was 1.26 ± 0.04 in S miR-365-treated tumor culture, compared to 0.98 ± 0.13 in scrambled-treated group).

## Discussion

In this study we identified and characterized two miRNAs with higher degree centrality in TNBC. In silico analysis of basal breast cancer profiles compared to normal breast epithelial tissue profile revealed that miR-135b is upregulated and miR-365 is downregulated in tumoral tissues compared to normal tissues. We validated these expressions in both cell lines and human BC tissue samples.

The integration of basal mRNA profile with miRNA profile revealed that miR-135b and miR-365 are involved in the control of several differentially expressed genes of TNBC, belonging to ‘Ethanol degradation IV’ and ‘Mismatch repair in eukaryotes’, and ‘Putrescine degradation III’, ‘Role of BRCA1 in DNA damage response’, and ‘Tryptophan degradation X’ pathways, respectively. A detailed analysis of the role of the two miRNAs within functional networks allowed us to suggest that both miRNAs are involved in the control of proliferation (predicted targets: *CHEK2*^19^, *E2F1*^20^, *RB1*^21^, or the genes of the FANC family^22^), migration and invasion. Indeed, the in vitro modulation of miR-135b, by transfection of As oligonucleotide, or of mimic miR-365, by transfection of S oligoucleotide, confirmed that both miRNAs are able to regulated cell proliferation rate, without inducing apoptosis of the cells (not shown). The antiproliferative effect is due to the regulatory role of both miRNAs on their targets. For miR-365, this could involve the regulation on putrescine degradation pathway, as suggested by^26^. The antiproliferative effect of miR-135b could be due to its activity on mismatch repair genes, such as in colorectal cancer^27^.

The experiments with miRNAs’ modulation also revealed that they are both involved in the control of invasion and migration, as suggested by the results of the wound healing and Boyden’s chamber. Liu et al.^28^ confirmed that miR-365 overexpression inhibits cell proliferation, migration and invasion, possibly by direct targeting *ADAM10*, a membrane-bound cell surface glycoprotein involved in cell adhesion of TNBC^29^. Among the possible target of miR-365, we found Ataxia Telangiectasia And Rad3-Related Protein (*ATR*) serine/threonine kinase, a mediator of the expression of several cell surface proteins of the extracellular matrix (ECM) required for adhesion and invasion of the cells^30^. The silencing of miR-135b or the overexpression of mimic miR-365 are able to reduce cellular mobility, perhaps due to the increased mesenchymal-to-epithelial (MET) differentiation process.

Indeed, both miRNAs are involved in the regulation of ALDH genes (*ALDH1A1*, *ALDH1A3* and *ALDH2* genes) belonging to the Tryptophan (Trp) degradation pathway, but also related to stemness. Cancer stem cell (CSC) populations have elevated ALDH activity^31^, and the expression of these genes is associated to chemoresistance in breast cancer^32^ and negative prognosis^33^. The downregulation of miR-365 in TNBC, could lead to the increase in ALDH genes expression guiding the cells to a more undifferentiated and aggressive phenotype. In our experiments, we observed a tendency towards the increase in epithelial marker expression (E-Cad) with miR-365 overexpression. The shift of As miR-135b- or S miR-365-treated cells toward epithelial phenotype is also suggested by the test of MS formation: the silencing of miR-135b or the increase of miR-365 led to a reduction of number and size of MS, compared to scramble treated cells. This partially confirm a possible role of miR-135b and miR-365 in controlling genes, involved in the maintenance of stemness. The study of Liu and colleagues demonstrated that the expression of *ALDH1A1* mRNA, one of predicted target mRNAs, in tumor tissues may be an independent predictor of TNBC outcome^34^.

Nowadays the treatment of TNBC is based on the combination of surgery removal of the cancer, radiation therapy and chemotherapy. Nevertheless, some patients don’t benefit from the chemotherapeutic treatment, leading to the development of relapse or metastasis in other tissues. Therefore, we also evaluated the possibility to use miRNA modulation in 3D structures, i.e. the organotypic culture of MDA-MB-231 cells. We succeeded in the reduction of miR-135b and in increase of miR-365 expression, suggesting a potential use of the two miRNA oligonucleotides in following experiments (that are beyond the objective of this work) aimed to deeper understand and validate these molecules and treatment strategy as new therapeutic drugs. Considering the anti-proliferative effects of the oligonucleotide treatment, their effect on blocking migration and invasion, and on favoring MET, their use could be helpful in counteracting the high metastatic potential of TNBC, alone or in combination with conventional therapy. No clinical trial is nowadays present on the use of miRNAs in TNBC treatment, although several publications propose their use as potential diagnostic or prognostic biomarkers^35,36^. The data need to be analysed in the light of a recent paper from Bao et al.^37^ who suggested that miR-135b-5p is downregulated in TNBC and this reduced expression would be important to increase proliferation and migration of the cell. These contradictory results could be partially justified by the fact that Bao et al. found miR-135b-5p downregulation in the comparison between TNBC and non-TNBC samples, correlated with the patient survival. On the contrary, our analysis identified miR-135b-5p as a possible regulator of a network of genes differentially expressed in TNBC versus normal tissues. We can’t exclude that miR-135b could be differentially expressed in non-TNBC compared to TNBC, as we didn’t perform this analysis on human samples.

Regarding miR-365, it has been already proposed as a tumor suppressor. Indeed, it has been found downregulated also in sera from early-stage breast cancer patients^38^. On the contrary, in HER2-positive breast cancer circulating exosomes, miR-365 was found to be significantly upregulated, suggesting that this miRNA could be expressed under the control of HER2 pathway^39^.

Although we propose miR135b and miR-365 as helpful biomarkers of TNBC diagnosis and for the development of new therapeutic tool, our study presents some limits: the first is linked to the fact that some sub-classifications of TNBC have been proposed. The paper of Perou highlighted that TNBC is also characterized by strong expression of basal markers such as cytokeratins 5, 6 and 17^40^. More recently, a study by Lehman et al.^41^ used histopathology and laser-capture micro-dissection to define 4 new subtypes of TNBC: basal subtypes 1 and 2 (BL1, BL2), characterized by elevated DNA damage response, higher expression of growth factor signaling pathways, glycolysis and gluconeogenesis; mesenchymal (M), enriched in components involved in cell motility, extracellular receptor interaction, and cell differentiation pathways; and luminal androgen receptor (LAR), enriched in hormonally regulated pathways such as steroid synthesis, porphyrin metabolism, and androgen/estrogen metabolism. In our analysis, we have considered what is defined as TNBC on the The Cancer Genome Atlas (TCGA) database, which included all the subtypes described before, without sub-classifying different molecular subtypes.

Our analyses possess the advantage to identify the network of genes that would be modulated by the miRNAs and not a single direct target since each of the two miRNAs analysed will be able to directly and indirectly modulate the entire network and no only the specific target. This approach will allow us to foresee a wider cellular outcome of their modulation. The identification of the specific direct targets will be done in the near future. Moreover, since the significant results of the analysis on human and organoid samples were obtained using a limited specimen number, further studies are needed for translating those miRNAs to clinical use. The use of miRNAs could be useful during the biopsy characterization phase. Besides, many miRNAs appear to be secreted in biological fluids, such as blood, plasma and serum. In this view, our study will pave the way for secreted miR-135b and miR-365 evaluation in different samples for their use in TNBC diagnosis or patient follow-up.

## Conclusions

By integrative in silico approach we identified two diagnostic miRNAs, miR-135b and miR-365, with a high degree centrality in basal breast cancer, involved in the control of proliferation, invasion and migration of tumoral cells and human samples. The modulation of these two miRNAs, obtained by oligonucleotide treatment, has an impact in their expression, leading to MET, characterized by a lower aggressive, epithelial phenotype. These two miRNAs could be proposed as tissue diagnostic molecules and as tools for the development of new therapeutic approaches for TNBC.

## Methods

### In silico analysis

#### RNA-Seq data

In order to obtain the gene profile altered in basal BC, we applied the computational approach on a IlluminaHiSeq RNASeqV2 dataset derived from TCGA. We used basal BC matched samples of mRNA and miRNAselecting 74 basal BC samples classified by PAM50 methods^16^. We used the available data from TCGA for the normal samples (NS), namely 113 NS for gene expression analysis and 87 NS for miRNA analysis. The expression levels of 1046 miRNAs and 15243 genes (excluding genes with a small variance) were considered. The data were pre-processed and normalized using the protocols described in TCGAbiolinks^14^.

#### Computational approach

The computational analysis consists of 6 steps: (1) identification of differentially expressed genes between basal and NS; (2) pathway enrichment analysis; (3) construction of classifier; (4) evaluation of classifier; (5) features selection able to classify with the best performance basal versus normal samples; (6) integration of miRNA in order to identify miRNAs regulating a pathway network in basal BC.

This computational approach was based on a Monte Carlo Cross-Validation^16^, dividing the original dataset into training (60%) and testing (40%) dataset. To avoid problems of unbalanced classes, we generated randomly an equal number of samples for each class, for both basal and NS.

### Cell culture

For in vitro studies, we used MDA-MB-231 cells and BT20 cell lines (ICLC, Genova, Italy), that are both referred as Basal, human, BC epithelial cell lines, and MCF10A cell line, as human, normal-like epithelial breast cells^42^. We maintained all the cell lines within a humidified atmosphere containing 5% CO_2_ at 37 °C. MDA-MB-231 cells were cultured in Advanced DMEM, while BT20 were culture in MEM cell culture medium (both Gibco, Life Technologies), added with 10% fetal bovine serum (FBS) (Lonza, Euroclone), 1% penicillin/streptomycin, 2 mM l-Glutamine following the manufacturer’s recommendation. MCF10A cells were cultured on Advanced DMEM with 10% FBS, 10 μg/ml insulin, 20 ng/ml hEGF, 0.5 μg/ml hydrocortisol, 2 mM l-Glutamine, 20 nM HEPES. Dulbecco Phosphate-Buffered Saline (D-PBS), and trypsin were obtained by Lonza (Euroclone).

### miRNA modulation

miRNA increase or decrease was obtained by transfection of 50 or 100 nM mimic or antisense oligonucleotide (Sigma Aldrich) with metafectene reagents (Biontex, Germany), following manufacturer’s instruction. The sequences for miRNA modulation were the following:

- miR-135b antisense (As): 5′- TCACATAGGAATGAAAAGCCATA-3′;
- miR-365-2-5p mimic (S): 5′-GGGACTTTCAGGGGCAGCT-3′.

A scramble sequence, non-codifying for any known miRNA, was used as a control of the experiments (Scramble miRNA: 5′-ATGATGTCCTTCTAGTACGCATC-3′).

All miRNAs were designed on the sequences from miRbase database ^43^.

### MTT assay

MDA-MB-231 cells were seeded in 96 wells at a confluence of 5000 cells/well. 24 h after seeding the cells were treated with mimic or antisense oligonucleotide for miRNA modulation. At 24, 48 and 72 h after treatment, the cells were stained with 50ug/ul of thiazolyl blue tetrazolium bromide (MTT) (Sigma Aldrich) for 4 h at 37 °C^44^. The cells were then lysed in dimethyl sulfoxide (DMSO; Euroclone, Italy) and the staining was quantified at 540 nm in FLUOstar Omega microplate reader (Euroclone). The presented results are the average ± sd of three independent experiments.

### RNA extraction and real time- quantitative PCR analysis

Total RNA was isolated using TRIzol reagent (Life Technologies) and it was reverse transcribed for the miRNA quantification, using MystiCq microRNA cDNA synthesis kit (Sigma Aldrich), following manufacturer’s recommendations and as already described in^44^. miRNAs were amplified in Eco real time-quantitative PCR (RT-qPCR) (Illumina, Euroclone) using Power Up Sybr green mix (Applied Biosystem), in combination with homemade designed primers (all from IDT, Tema Ricerca, Italy): miR-365-2-5p Fw primer: 5′-GGGACTTTCAGGGGCAGCT-3′; miR-135b Fw primer 5′-TATGGCTTTTCATTCCTATGTGA-3’.

For each RT-qPCR analysis, the results are presented as values of relative expression 2^-ΔC(T) or as fold change expression (2^-ΔΔCt) using real-time qPCR (Eco, Illumina, Euroclone, Italy) normalizing the results on the expression of a housekeeping miRNA, miR-103-3p (5′-AGCAGCATTGTACAGGGCTATGA-3′) or the positive control present in the MystiCq microRNA cDNA synthesis kit.

### Cell migration: wound healing

To study directional cell migration, we performed a wound-healing study. The MDA-MB-231cells were seeded at a confluence of 150–200,000/well in 24-well plate (Euroclone). After 48 h, necessary to reach 80% confluence, a scratch was performed in the middle of each well^45^. After washing the cells, new medium was added, and miRNAs were modulated, as described before. Pictures of each well were taken every 24 h. The effect on cell migration was quantified by ImageJ software^46^. The quantification of the wound was performed calculating the area of the scratch at 24 h normalized on the area in the same point of the well at time 0. This means that if a treatment reduced the capability of the cells to restore and fill up the wound, the ratio of the area 24 h/0 h should increase in respect to untreated or scramble treated cells, following the method described in^46^. Experiments were performed in triplicate (*n* = 9). A *t* test was calculated among untreated, scramble-treated, S miR-365-2-5p, or As miR-135b-treated MDA-MB-231 cells.

### Cell invasion: Boyden’s chamber

A standard Boyden’s chamber test was used for the invasion study. The test was performed and quantified as described previously^44^.

### Mammosphere formation test

For mammosphere (MS) generation, MDA-MB-231 cells were cultured on non-adherent plates (Corning, Sigma, Italy) at 100 single cells/ml. The culture medium was DMEM supplemented with B27 (Invitrogen), 20 ng/ml EGF (ThermoFisher Scientific, Life Technologies, Italy), 20 ng/ml bFGF and 4 μg/ml heparin (both Sigma, Italy) added, when required, with 50 nM scramble, As miR-135b or S miR-365 oligonucleotide (IDT, Tema Ricerca; Italy) every 2 days. Pictures of MS growth were taken every 2 days. We counted how many surface cells are present for every 10 MS (picture 20 ×) ^47^.

### BC human tissue samples validation

Eight samples of TNBC human tissue and corresponding non-tumoral clear margins were selected from the collection of the “Bruno Boerci” Oncological Biobank for research applications (Istituti Clinici Scientifici Maugeri IRCCS, Pavia), a biobank certified to the ISO 9001:2015 international standard and member of BBMRI.it (the italian node of the BBMRI-ERIC, Biobanking and BioMolecular resources Research Infrastructure—European Research Infrastructure Consortium)^44^. Surgical resections were performed from 2011 to 2013 at the Breast Unit of Istituti Clinici Scientifici Maugeri IRCCS, Pavia, Italy. Fresh tumor and normal breast specimens from surgical resections have been collected immediately after breast-conserving surgery or total mastectomy performed at Breast Unit of Istituti Clinici Scientifici Maugeri IRCCS, Pavia, Italy. A written and oral informed consent to participate in the present protocol was informed and obtained from each patient. This study was approved by the Maugeri Ethical Committee. This study was performed in accordance with the relevant guidelines and regulations. Tissues are snap-frozen in liquid nitrogen within 30–60 min of surgery excision and stored at − 80 °C at the “Bruno Boerci” Oncological Biobank until further use, according to the guidelines of the European research infrastructure for biobanking BBMRI-ERIC (the Bruno Boerci Oncological Biobank Standard Operating Procedure is certified ISO 9001:2015 and ISO 20387:2018 standards).

At the time of collection, TNBC characterization was assessed through immunohistochemical analysis (IHC) by the Pathology Service (Istituti Clinici Scientifici Maugeri IRCCS, Pavia), according to the clinical guidelines on BC (ASCO—American Society of Clinical Oncology). IHC characterization of the tumor samples is shown in Table 2 and supplementary Table 1. The samples were used for the isolation of total RNA and miRNA analysis in real-time quantitative PCR (as described in the methods below).

### Organotypic tissue culture and in vitro treatment

Primary tumors obtained from Athymic Nude-Foxn1 nu/nu mice implanted with MDA-MB-231 cells were isolated in sterile condition. Slices were cut at the vibratome (50 nu each) and put in culture on Millicent supports (Millipore) in the Epicult Medium, supplemented by 10% FBS, 1% Penicillin/Streptomycn, 100 μg/ml kanamycin. Fifty nanomolar mimic miR365 or As miR135b were added to the medium in the presence of GenomOne-Neo Ex (CosmoBio) transfection reagent, following manufacturer’s protocol. The organotypic-like culture tissues were maintained in culture for 48–72 h, as suggested by^48^; RNA was then isolated and RT-qPCR analyses for miR-135b and miR-365 were performed, as described before. Animal experiments were carried out in compliance with the institutional guidelines for the care and use of experimental animals (European Directive 2010/63/UE and the Italian law 26/2014) and with the ARRIVE guidelines (https://arriveguidelines.org), authorized by the Italian Ministry of Health and approved by the Animal Welfare Organisation of the University of Milan.

### Ethics approval and consent to participate

As reported in the section “Methods”, TNBC human tissue and corresponding non-tumoral clear margins were selected from the collection of the “Bruno Boerci” Oncological Biobank for research applications (Istituti Clinici Scientifici Maugeri IRCCS, Pavia), a biobank certified to the ISO 9001:2015 international standard and member of BBMRI.it (the Italian node of the BBMRI-ERIC, Biobanking and BioMolecular resources Research Infrastructure—European Research Infrastructure Consortium). Surgical resections were performed from 2011 to 2013 at the Breast Unit of Istituti Clinici Scientifici Maugeri IRCCS, Pavia, Italy. Written informed consents from patients were obtained in advance and tissue samples were collected, processed and stored at − 80 °C as snap-frozen aliquots immediately after surgery, according to the best practices in biobanking (ISO 9001:2015 and ISO 20387:2018 standards). At the time of collection, TNBC characterization was assessed through immunohistochemical anlysis (IHC) by the Pathology Service (Istituti Clinici Scientifici Maugeri IRCCS, Pavia), according to the clinical guidelines on BC (ASCO—American Society of Clinical Oncology). IHC characterization of the tumor samples is shown in Table 1. The samples were used for the isolation of total RNA and miRNA analysis in real-time PCR (as described in the “Methods” below).

## Supplementary Information

Supplementary Table S1.

Supplementary Figure S1.

Supplementary Figure S2.

Supplementary Figure S3.

Supplementary Figure S4.

## Data Availability

In order to obtain the gene profile altered in TNBC, we applied the computational approach on a IlluminaHiSeq RNASeqV2 dataset derived from The Cancer Genome Atlas (TCGA), https://www.cancer.gov/tcga.
