# Supplementary figures and images for: Triple negative aggressive phenotype controlled by miR-135b and miR-365: new theranostics candidates
Source: Sci Rep. 2021 Mar 22;11:6553. doi: 10.1038/s41598-021-85746-w (PMC7985188; doi:10.1038/s41598-021-85746-w)

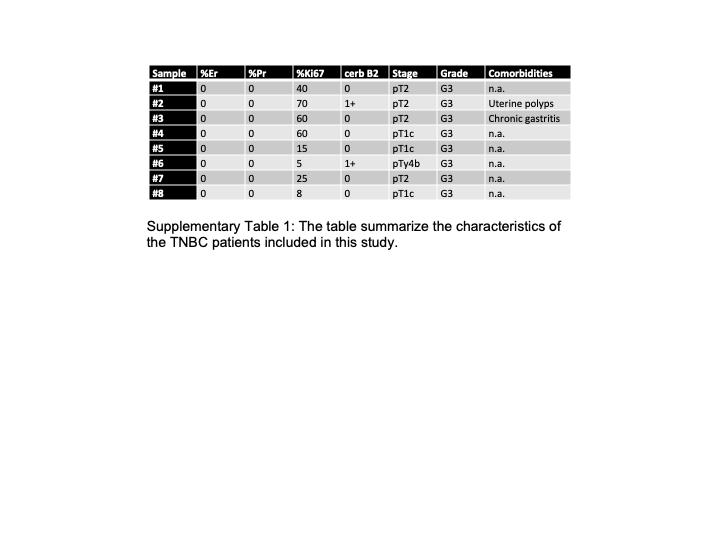

Supplement: Supplementary file 1 — Supplementary Table S1. [file 41598_2021_85746_MOESM1_ESM.tiff]

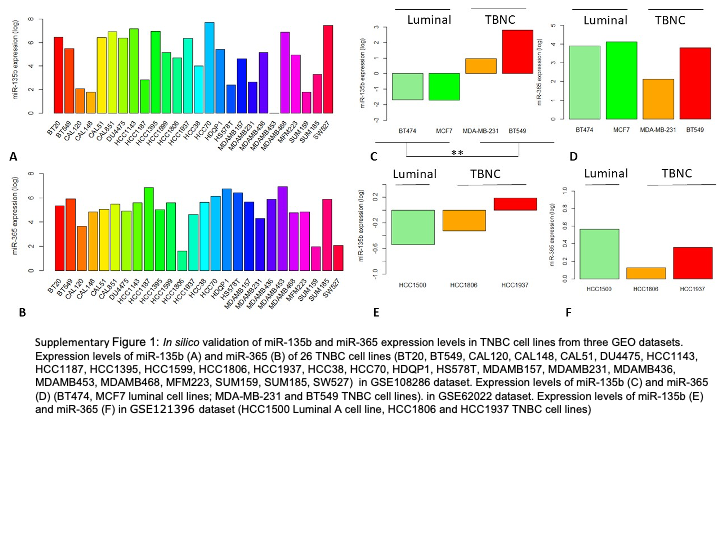

Supplement: Supplementary file 2 — Supplementary Figure S1. [file 41598_2021_85746_MOESM2_ESM.tiff]

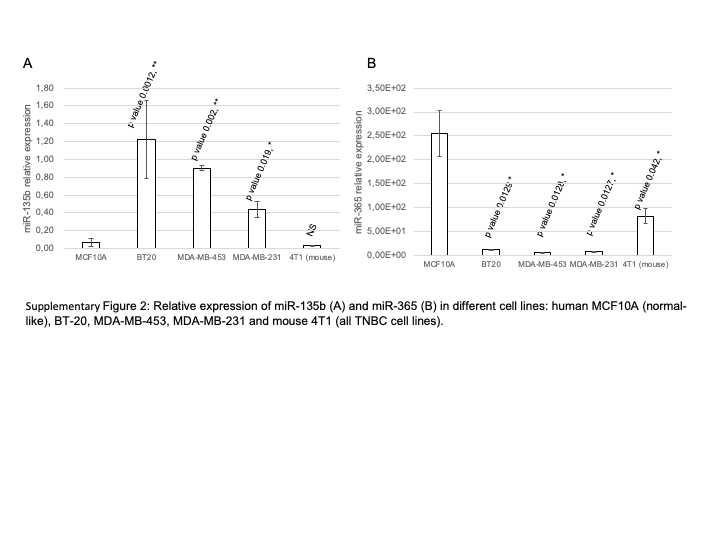

Supplement: Supplementary file 3 — Supplementary Figure S2. [file 41598_2021_85746_MOESM3_ESM.tiff]

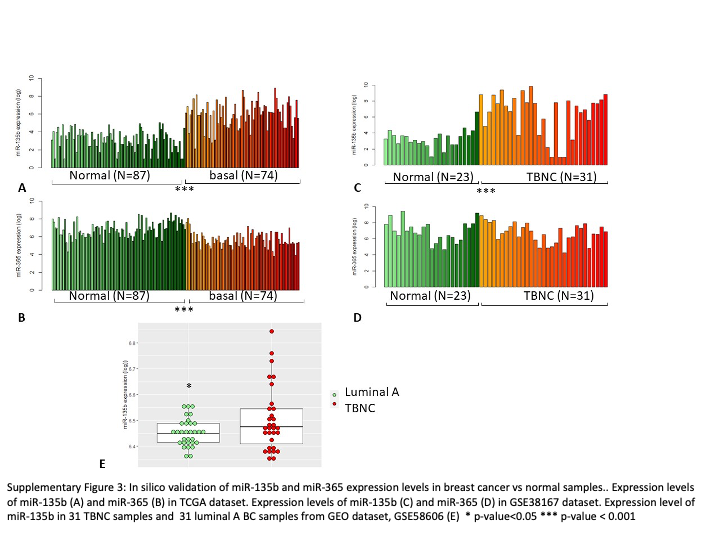

Supplement: Supplementary file 4 — Supplementary Figure S3. [file 41598_2021_85746_MOESM4_ESM.tiff]

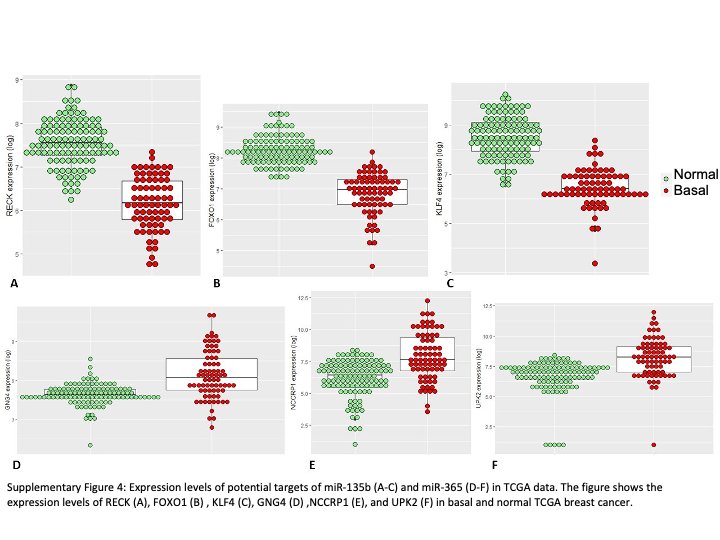

Supplement: Supplementary file 5 — Supplementary Figure S4. [file 41598_2021_85746_MOESM5_ESM.tiff]
